# A new genus for a rare African vespertilionid bat: insights from South Sudan

**DOI:** 10.3897/zookeys.285.4892

**Published:** 2013-04-05

**Authors:** DeeAnn M. Reeder, Kristofer M. Helgen, Megan E. Vodzak, Darrin P. Lunde, Imran Ejotre

**Affiliations:** 1 Department of Biology, Bucknell University, Lewisburg, Pennsylvania, 17837, USA; 2 Division of Mammals, National Museum of Natural History, Smithsonian Institution, P.O. Box 37012, Washington, DC 20013-7012, USA; 3 Department of Biological Sciences, Islamic University in Uganda, Mbale, Uganda

**Keywords:** *Glauconycteris superba*, *Glauconycteris poensis*, *Glauconycteris curryae*, *Niumbaha* gen. nov., Badger Bat, South Sudan, Description

## Abstract

A new genus is proposed for the strikingly patterned African vespertilionid “*Glauconycteris” superba* Hayman, 1939 on the basis of cranial and external morphological comparisons. A review of the attributes of a newly collected specimen from South Sudan (a new country record) and other museum specimens of “*Glauconycteris” superba* suggests that “*Glauconycteris” superba* is markedly distinct ecomorphologically from other species classified in *Glauconycteris* and is likely the sister taxon to *Glauconycteris* sensu stricto. The recent capture of this rarely collected but widespread bat highlights the need for continued research in tropical sub-Saharan Africa and in particular, for more work in western South Sudan, which has received very little scientific attention. New country records for *Glauconycteris* cf. *poensis* (South Sudan) and *Glauconycteris curryae* (Gabon) are also reported.

## Introduction

In 1939 Hayman described a new vespertilionid bat from the Belgian Congo (now Democratic Republic of the Congo), noting that it was “one of the most striking discoveries of recent years’’ (Hayman 1939). He placed this species in the genus *Glauconycteris* Dobson, 1875, aptly erecting the specific name *superba* for its spectacularly bold black and white color pattern. Since that time, only a few specimens of this species have been collected. Our capture of a parous female in July 2012 in southwestern South Sudan represents a new country record for this poorly known bat, extending its range eastward. The only species of *Glauconycteris* previously reported from South Sudan is *Glauconycteris variegata* (Koopman 1975, McLellen 1986).

*Glauconycteris*, originally described by Dobson (1875) as a subgenus of *Chalinolobus*, is found in Africa south of the Sahara and is currently recognized as having 12 species (Simmons 2005, Rambaldini 2010). Its species are restricted, more or less, to forested tropical areas and savanna woodlands. While one or two species of *Glauconycteris* are widely distributed, many are poorly known and relatively poorly represented in museum collections. *Glauconycteris* bats are characterized by a highly distinctive combination of traits, including variable patterns of spots and stripes on the body, reticulated wings, and an extremely shortened muzzle and toothrow. Within the large family Vespertilionidae, *Glauconycteris* is classified in the subfamily Vespertilioninae, tribe Nycticeiini (Hoofer and Van Den Bussche 2003) and forms a clade with *Lasionycteris*, *Nycticeius*, *Arielulus*, *Eptesticus*, and *Scotomanes* (Roehrs et al. 2011). Hayman (1939) placed *superba* in *Glauconycteris* on the basis of its boldly patterned markings, dental formula, and properties of the incisors (Rosevear 1965; Rambaldini 2010).

Close examination of our 2012 South Sudan specimen relative to other specimens of *Glauconycteris superba* and of other *Glauconycteris* species indicates that, while this taxon is probably closely related to species of *Glauconycteris*, it lacks many of the most notable specializations of that genus, and we suggest that it is sufficiently and remarkably different from other vespertilionids as to warrant placement in a unique genus.

## Materials and methods

**Figure 1.**
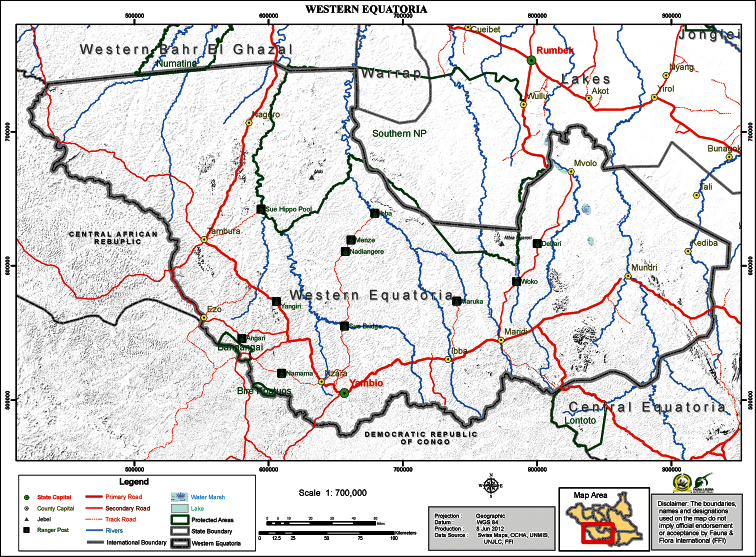
Map of Western Equatoria State, South Sudan. Location of the Bangangai Game Reserve (and the neighboring Bire Kpatuos Game Reserve) and other protected areas shown.

Field work was conducted in Bangangai Game Reserve, Western Equatoria State in the new country of South Sudan in July 2012 (Fig. 1). Bats, including the single “*Glauconycteris*” *superba* specimendescribed below and two other species of *Glauconycteris*, were captured in single ground-height or triple-high mist-nets and euthanized by isoflurane overdose. Tissue samples (liver and muscle) were collected and flash frozen in liquid nitrogen. Specimens were either formalin fixed and then transferred to ethanol with skulls extracted or were prepared as skins, skulls, and skeletal material. Field work was approved by the Internal Animal Care and Use Committee of Bucknell University and by the South Sudanese Ministry for Wildlife Conservation and Tourism.

A comparative analysis included data from our 2012 specimens, a few South Sudan specimens from our earlier expeditions, data from the three previously collected specimens of “*Glauconycteris” superba* and data from museum specimens as noted below. Measurements were taken with rulers (ears) or dial calipers (all other measurements). External and osteological characters examined are based largely upon Eger and Schlitter (2001) (see Table 1). Differences in wing-tip length between species of *Glauconycteris* and “*Glauconycteris” superba* were determined with a t-test and both principal components analysis (PCA) and t-tests were performed on cranial and dental data. PCA was performed using the combination of cranial and dental measurements indicated in tables and in the text. All measurement values were transformed to natural logarithms prior to multivariate analysis. Principal components were extracted from a covariance matrix. Variables for multivariate analyses were selected judiciously to maximize sample sizes for comparison by allowing for inclusion of partially broken skulls in some cases. The software programs Statistica 8.0 (Statsoft Inc., Tulsa, Oklahoma, USA) and SPSS Statistics 19.0 @ 2010 (IBM Corporation, Somers, NY, USA) were used for all analytical procedures.

**Table 1.** Definition of external, craniodental, and mandibular measurements used in this study.<br/>

**Measurement**	**Definition**
Forearm length (FA)	Distance from the elbow (tip of the olecranon process) to the wrist (including the carpals).
Metacarpal length (ML-III, -IV, -V)	Distance from the joint of the wrist (carpals) with the 3rd metacarpal to the metacarpophalangeal joint of the 3rd digit; same for 4th and 5th digits.
Phalangeal length (1PL, 2PL)	1PL: Distance from the metacarphophalangeal joint of each respective digit (DI, DII, DIII) to the phalangeal joint. 2 PL: Distance from the phalangeal joint to the tip of the bone (cartilage tip not included).
Greatest length of skull (GLS)	Greatest distance from the occiput to the anteriormost point on the premaxilla.
Condyloincisive length (CIL)	Distance between a line connecting the posteriormost margins of the occipital condyles and the anteriormost point on the upper incisors.
Condylocanine length (CCL)	Distance between a line connecting the posteriormost margins of the occipital condyles and the anteriormost surfaces of the upper canines.
Palatal length	Distance from the posterior palatal notch to the anteriormost border of the incisive alveoli.
Zygomatic breadth (ZB)	Greatest breadth across the zygomatic arches.
Mastoid width	Greatest breadth at the mastoid processes.
Breadth of braincase (BBC)	Greatest breadth of the globular part of the braincase, excluding mastoid and paraoccipital processes.
Height of braincase (HBC)	Distance from basisphenoid and basioccipital bones to top of braincase on either side of sagittal crest.
Interorbital width	Distance between orbits measured below lachrymal processes.
Postorbital process width (POP)	Width across postorbital processes.
Postorbital constriction (POC)	Least distance between orbits.
Width across M^3^ (M^3^-M^3^)	Greatest width of palate across labial margins of the alveoli of M^3^s.
Maxillary toothrow length (C-M^3^)	Distance from anteriormost surace of the upper canine to the posteriormost surface of the crown of M^3^.
Width at upper canines (C-C)	Width between labial alveolar borders of upper canines.
Greatest length of mandible	Distance from midpoint of condyle to the anteriormost point of the dentary, including the incisors.
Mandibular toothrow length (c-m_3_)	Distance from posterior alveolar border of m_3_ to the anterior alveolar border of lower canine.
Height of the upper canine	Greatest height of the upper canine from point immediately dorsal to cingulum to end of tooth (not taken if tooth too worn).
Thickness of the upper canine	Greatest anterior-posterior thickness of the upper canine.
Width M^3^ (WM^3^)	Greatest lateral-medial width of last tooth (M^3^).
Width M^2^ (WM^2^)	Greatest lateral-medial width of second to last tooth (M^2^).
Mid rostrum length (MRL)	Length of a medial line from the inflexion point at the rostrum/braincase to posterior point of emargination in the upper palate.
I-M^2^ alv	Length from anterior alveoli of incisors to posterior alveoli of second to last tooth (M^2^).

## Taxonomy

### Niumbaha

Reeder et al. gen. n.

urn:lsid:zoobank.org:act:EDF16BEE-0749-41BC-AE19-BAE130BE58F8

http://species-id.net/wiki/Niumbaha

**Figure 2.**
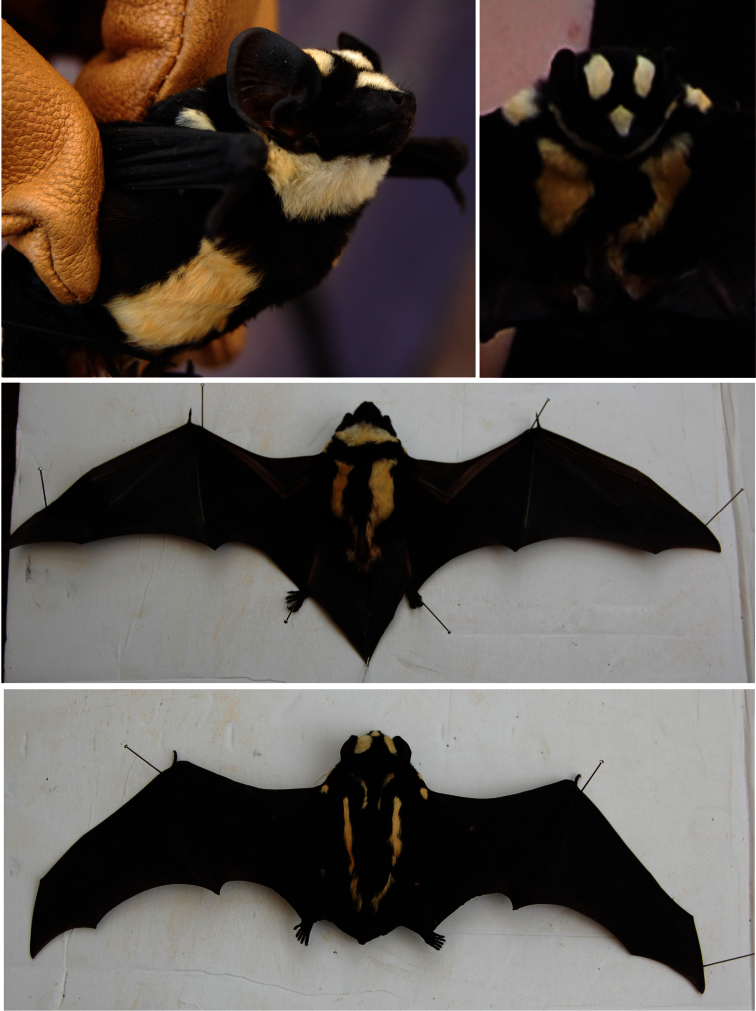
Photographs of *Niumbaha superba* live and as a freshly prepared specimen. Top photos show profile and anterior view, with ventral and dorsal images below.

Figures 2 [Fig F3] [Fig F4] [Fig F5] –6

#### Etymology.

The name is the Zande word for ‘rare/unusual’. This name was chosen because of the rarity of capture for this genus, despite its wide distribution throughout West and Central Africa, and for the unusual and striking appearance of this bat. Zande is the language of the Azande people, who are the primary ethnic group in Western Equatoria State in South Sudan (where our recent specimen was collected). The homeland of the Azande extends westwards into Democratic Republic of the Congo, where *superba* has also been collected (the holotype and another recent capture), and into southeastern Central African Republic. Gender: feminine.

#### Type species.

*Glauconycteris superba* Hayman, 1939; by monotypy.

#### Diagnosis.

**Figure 3. F3:**
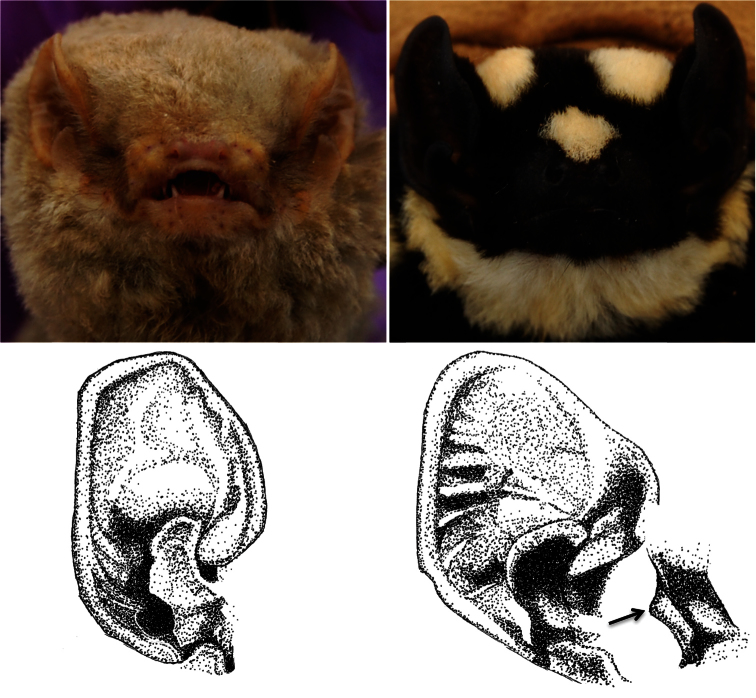
Contrasting facial aspects for *Glauconycteris* cf. *poensis* (left) and *Niumbaha superba* (right). Top panels show differences in nostril shape and orientation from photographs of live bats, bottom drawings show difference in ear and tragus structure. *Glauconycteris poensis* and *Niumbaha superba* are the type species of *Glauconycteris* and *Niumbaha*.

**Figure 4. F4:**
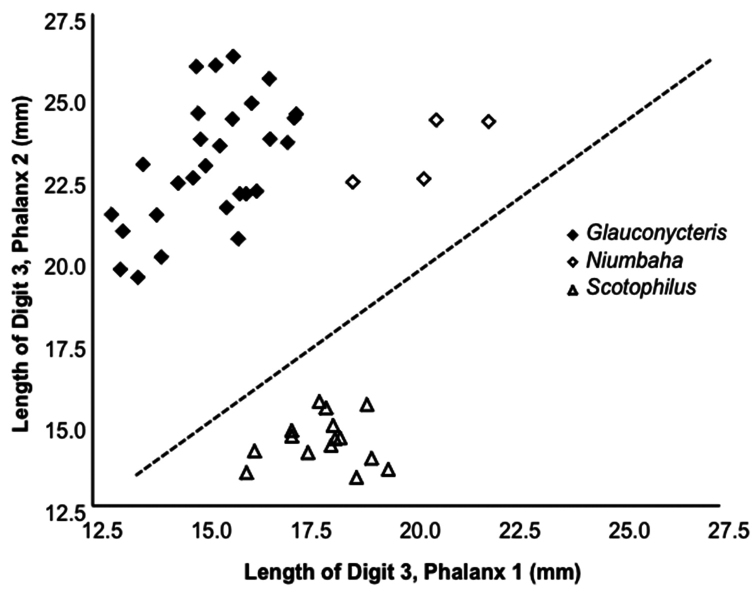
Length of the 2^nd^ phalanx (2PL) of the 3^rd^ digit vs. the 1^st^ phalanx (1PL) of the 3^rd^ digit. Several species of *Glauconycteris* are shown (closed diamond), as is *Niumbaha superba* (open diamond), and for comparison, two species of *Scotophilus* (open triangle; a ‘typical’ African vespertilionid bat). The ratio of 2PL/1PL is significantly greater in *Glauconycteris* than in *Niumbaha* (with a theoretical 1:1 ratio indicated by the dashed line). Data as reported in Table 2.

**Figure 5. F5:**
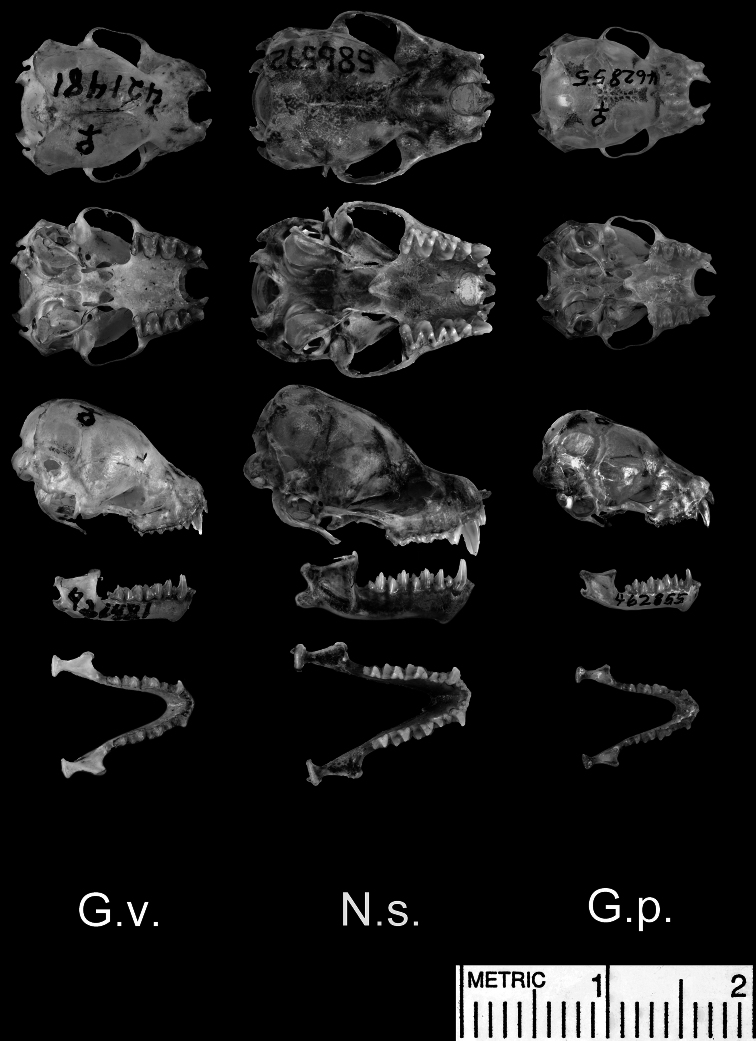
Dorsal and ventral views of the cranium, lateral views of the cranium and mandible, and dorsal view of the mandible. Species shown include *Glauconycteris variegata* (G.v.; a relatively large species of *Glauconycteris*, which nearly matches *Niumbaha superba* in linear body size, but not in skull size); *Niumbaha superba* (N.s.; the type species of *Niumbaha*), and *Glauconycteris poensis* (G.p., the type species of *Glauconycteris*).

Among vespertilionids, *Niumbaha* bears closest comparison with species of *Glauconycteris* (the type species of which is *Glauconycteris poensis*), to which it is apparently closely related, but it has a considerably larger skull and is more strikingly patterned compared to any member of *Glauconycteris* (its patterning most closely approaching the Asian vespertilionid genus *Scotomanes*). It lacks various of the most exaggeratedly derived traits (specializations) that uniquely unite the species of *Glauconycteris* among African vespertilionids, including the excessively foreshortened rostrum, moderately to highly reduced relative canine size, and very elongate wing tips (second wing phalanxes) of *Glauconycteris* (Rosevear 1965). Externally, *Niumbaha* is immediately distinguished from all other African vespertilionid bats by its distinct coloration pattern, including pale yellow spots and stripes on an otherwise dark black pelage (Fig. 2, Fig. 3, and detailed descriptions below). While Hayman (1939:222) noted that, “in general form *Glauconycteris superba* does not differ from other *Glauconycteris*,” we find that most external features are in fact different from *Glauconycteris sensu stricto*. The ears of *Niumbaha* are more robust and subquadrangular, contain a larger free lobe at the inner margin, and contain a more strongly curved tragus than *Glauconycteris* (Fig. 3). The muzzle of *Niumbaha* is more robust than *Glauconycteris sensu stricto* and contains nostrils that open more to the front than to the side (Fig. 3). The wingtips in *Niumbaha* are longer than in most other African vespertilionids in that phalanx 2 of the third digit is longer than phalanx 1, yet remain considerably shorter than in the characteristically long-wingtipped *Glauconycteris* (ratio of Ph2/Ph1 in *Niumbaha*, at 1.15 ± 0.05 SD, is significantly shorter than *Glauconycteris*, at 1.51 ± 0.12 SD; Fig. 4). *Niumbaha* shares its dental formula with *Glauconycteris*, at 2.1.1.3/3.1.2.3 = 32, but is overall significantly larger than species of *Glauconycteris* in all characters, with a total skull length of greater than 16.0 mm (Table 2; Fig. 5). While the rostrum of *Glauconycteris* is short and generally rises in an even plane from the incisors to the occiput, the frontal region of the skull in *Niumbaha* is excavated or ‘hollowed out’, with the upper surface of the longer rostrum largely flat and roughly parallel to the upper toothrows (see Fig. 5). Additionally, the skull is relatively less broad and less domed and more elongate than in *Glauconycteris* (indicated by ratios of the mastoid width, breadth of the braincase, height of the braincase, and zygomatic breadth to the greatest length of the skull (Table 2)), although the anterior portion of the rostrum is relatively broader (indicated by the ratio of the width at the upper canines to the width at the last molar (M^3^-M^3^)).

**Table 2.** Selected measurements (in mm) of *Niumbaha superba* and several *Glauconycteris* and *Scotophilus* species. Summary statistics (mean and standard deviation), observed range and sample size of measurements are given for each species. See Table 1 for definition of measurement abbreviations and see methods for list of specimens examined.

**Character**	Niumbaha superba*	***Glauconycteris alboguttata***	***Glauconycteris argentata***	***Glauconycteris beatrix***	***Glauconycteris curryae***	***Glauconycteris humeralis***	***Glauconycteris poensis***	***Glauconycteris* cf. *poensis***	***Glauconycteris variegata***	***Scotophilus leucogaster***	***Scotophilus viridis***
**ML-III**											
*X*‾ ± *SD*	44.7 ± 2.3	39.8	41.7 ± 0.7	37.8 ± 1.9	35.0	-	38.4 ± 1.3	42.3 ± 1.1	42.3 ± 1.3	50.8 ± 1.4	46.8 ± 2.5
Min-max	42.0–47.4	-	40.8–42.3	35.2–39.6	-	-	36.4–39.6	40.8–44.3	40.5–44.1	48.6–52.4	41.5–50.1
*n*	4	1	4	4	1	-	5	5	8	8	9
**DIII-1PL**											
*X*‾ ± *SD*	20.4 ± 1.3	16.0	15.6 ± 0.6	13.4 ± 0.9	13.6	-	13.9 ± 0.9	15.6 ± 0.7	16.6 ± 0.6	18.6 ± 0.5	17.1 ± 1.1
Min-max	18.7–22.0	-	15.0–16.3	12.3–14.5	-	-	12.9–15.2	14.9–16.7	15.7–17.4	18.1–19.6	15.7–19.2
*n*	4	1	4	4	1	-	5	5	8	8	9
**DIII-2PL**											
*X*‾ ± *SD*	23.4 ± 1.1	22.0	25.7 ± 0.6	21.3 ± 1.6	19.5	-	21.3 ± 1.0	24.1 ± 0.8	22.8 ± 1.4	14.6 ± 0.8	13.9 ± 1.2
Min-max	22.4–24.3	-	24.8–26.2	19.7–22.9	-	-	20.1–22.9	22.5–25.6	20.7–24.5	13.4–15.5	11.9–15.7
*n*	4	1	4	4	1	-	5	5	8	8	9
**ML-IV**											
*X*‾ ± *SD*	43.4 ± 2.5	35.8	39.1 ± 1.1	34.1 ± 1.8	32.5	-	34.9 ± 1.1	39.3 ± 1.4	40.8 ± 1.3	48.9 ± 1.6	45.6 ± 2.3
Min-max	40.6–46.4	-	37.7–40.3	31.6–36.0	-	-	33.6–36.4	37.0–41.8	39.4–42.5	46.4–50.6	41.4–48.9
*n*	4	1	4	4	1	-	5	5	8	8	9
**DIV-1PL**											
*X*‾ ± *SD*	13.5 ± 1.2	11.8	11.7 ± 0.4	10.1 ± 0.7	8.8	-	11.1 ± 0.4	11.6 ± 0.7	12.7 ± 0.9	13.7 ± 1.1	13.5 ± 1.0
Min-max	12.2–15.0	-	11.4–12.1	9.1–10.8	-	-	10.5–11.4	10.8–12.2	11.2–13.9	11.3–15.1	12.1–14.8
*n*	4	1	4	4	1	-	5	5	8	8	9
**DIV-2PL**											
*X*‾ ± *SD*	10.1 ± 0.9	10.8	11.8 ± 0.6	11.5 ± 1.1	12.0	-	10.8 ± 0.9	11.6 ± 0.8	12.3 ± 0.8	10.2 ± 0.3	9.1 ± 0.9
Min-max	9.0–10.8	-	11.2–12.4	10.1–12.6	-	-	9.9–11.9	10.6–12.5	10.9–13.5	9.8–10.7	7.2–10.2
*n*	4	1	4	4	1	-	4	5	8	8	9
**ML-V**											
*X*‾ ± *SD*	38.8 ± 2.8	31.2	35.5 ± 0.9	32.5 ± 1.2	29.9	-	32.1 ± 0.8	35.5 ± 0.8	38.6 ± 1.5	47.1 ± 1.6	42.9 ± 1.9
Min-max	35.5–42.0	-	34.3–36.3	31.3–34.2	-	-	30.6–32.8	33.7–37.0	36.1–40.4	43.9–49.4	39.5–45.8
*n*	4	1	4	4	1	-	5	5	8	8	9
**DV-1PL**											
*X*‾ ± *SD*	8.8 ± 1.2	9.6	9.6 ± 0.4	9.4 ± 0.4	8.4	-	9.8 ± 0.5	10.3 ± 1.0	10.6 ± 0.6	10.2 ± 0.9	9.0 ± 0.6
Min-max	7.6–10.4	-	9.2–10.2	8.8–9.7	-	-	9.1–10.3	9.1–11.4	9.7–11.4	8.9–11.6	7.9–9.6
*n*	4	1	4	4	1	-	5	5	8	8	9
**DV-2PL**											
*X*‾ ± *SD*	7.5 ± 0.7	7.8	8.7 ± 0.6	7.4 ± 0.5	7.6	-	8.1 ± 0.5	7.8 ± 0.4	8.3 ± 0.9	6.4 ± 0.3	6.4 ± 0.7
Min-max	6.8–8.2	-	8.3–9.5	6.8–7.9	-	-	7.5–8.8	6.7–8.4	7.2–9.8	5.9–6.9	5.7- 7.4
*n*	4	1	4	4	1	-	5	5	8	8	9
**DIII-2PL/1PL**											
*X*‾ ± *SD*	1.1 ± 0.1	1.4	1.7 ± 0.1	1.6 ± 0.1	1.4	-	1.5 ± 0.1	1.6 ± 0.1	1.4 ± 0	0.8 ± 0.1	0.8 ± 0.01
Min-max	1.1–1.2	-	1.5–1.7	1.5–1.7	-	-	1.4–1.7	1.5–1.6	1.3–1.4	0.7–0.9	0.7–0.9
*n*	4	1	4	4	1	-	5	5	8	8	9
**DIV-2PL/1PL**											
*X*‾ ± *SD*	0.8 ± 0.1	0.9	1.0 ± 0.0	1.1 ± 0.1	1.4	-	1.0 ± 0.1	1.0 ± 0.1	1.0 ± 0.1	0.8 ± 0.1	0.7 ± 0.1
Min-max	0.7–0.8	-	1.0–1.1	1.0–1.3	-	-	0.9–1.1	0.9–1.1	0.9–1.1	0.7–0.8	0.6–0.8
*n*	4	1	4	4	1	-	4	5	8	8	9
**GLS**											
*X*‾ ± *SD*	16.8 ± 0.6**	13.3	12.7 ± 0.3	11.4 ± 0.2	12.2	11.1 ± 0	12.3 ± 0.3	-	13.9 ± 0.3	20.5 ± 0.3	18.0 ± 0.7
Min-max	16.2–17.4	13.2–13.4	12.0–13.3	11.2–11.6	-	11.1–11.1	12.0–12.7	-	13.4–14.4	20.1–20.9	17.0–18.4
*n*	4	2	12	3	1	3	6	-	23	7	4
**CIL**											
*X*‾ ± *SD*	15.6 ± 0.4**	12.8	12.3 ± 0.3	11.1 ± 0.3	11.1	10.9 ± 0.1	11.9 ± 0.3	-	13.3 ± 0.3	18.0 ± 0.2	16.3 ± 0.5
Min-max	15.4–16.2	12.7–12.9	11.7–12.5	10.9–11.5	-	10.8–11.0	11.5–12.4	-	12.8–13.8	17.7–18.3	15.6–16.6
*n*	4	2	12	3	1	3	6	-	23	7	4
**CCL**											
*X*‾ ± *SD*	16.0	12.4	11.9 ± 0.4	10.9 ± 0.3	10.7	10.8 ± 0.3	11.5 ± 0.3	-	12.9 ± 0.3	17.5 ± 0.3	15.8 ± 0.4
Min-max	-	12.4–12.4	11.0–12.2	10.7–11.2	-	10.5–11.0	11.1–12.0	-	12.3–13.4	17.1–17.9	15.3–16.3
*n*	1	2	13	3	1	3	7	-	24	7	4
**Palatal length**											
*X*‾ ± *SD*	5.9 ± 0.4**	5.3	4.8 ± 0.2	4.4 ± 0.1	-	4.6 ± 0.2	4.8 ± 0.5	-	5.2 ± 0.3	7.1 ± 0.1	6.5 ± 0.5
Min-max	5.5–6.5	5.1–5.5	4.4–5.3	4.3–4.5	-	4.4–4.8	4.4–5.5	-	4.8– 6.0	6.9–7.3	6.1–7.2
*n*	4	2	13	3	-	3	4	-	22	7	4
**ZB**											
*X*‾ ± *SD*	11.4 ± 0.5**	9.5	9.0 ± 0.2	8.3 ± 0.2	8.5	8.2	8.6 ± 0.2	-	10.2 ± 0.3	13.1 ± 0.4	12.0 ± 0.4
Min-max	11.0–11.9	9.4–9.5	8.6–9.2	8.1–8.4	-	8.0–8.3	8.4–8.9	-	9.5–10.9	12.7–13.8	11.5–12.3
*n*	4	2	10	3	1	2	7	-	23	7	4
**Mastoid width**											
*X*‾ ± *SD*	9.6 ± 0.2**	8.4	8.2 ± 0.3	7.5 ± 0.1	7.3	7.3 ± 0.2	7.7 ± 0.2	-	8.9 ± 0.2	11.5 ± 1.0	10.2 ± 0.4
Min-max	9.5–9.9	8.4 – 8.4	7.9–8.5	7.4–7.6	-	7.1–7.4	7.5–8.0	-	8.4–9.4	9.3–12.3	9.6–10.5
*n*	4	2	12	3	1	3	7	-	23	7	4
**BBC**											
*X*‾ ± *SD*	8.7 ± 0.3**	7.8	7.6 ± 0.2	6.9 ± 0.1	6.8	6.8 ± 0.1	7.2 ± 0.3	-	8.0 ± 0.2	9.2 ± 0.2	8.3 ± 0.2
Min-max	8.5–9.0	7.7–7.9	7.4–8.0	6.9–7.0	-	6.7–6.9	6.8–7.4	-	7.6–8.4	8.8–9.4	8.1–8.5
*n*	4	2	12	3	1	3	7	-	24	7	4
**HBC**											
*X*‾ ± *SD*	6.9 ± 0.3**	5.8	5.7 ± 0.2	5.1 ± 0.1	4.9	5.1 ± 0.1	5.4 ± 0.2	-	6.0 ± 0.1	8.2 ± 0.3	6.8 ± 0.5
Min-max	6.6–7.3	5.8 – 5.8	5.5–6.0	4.9–5.2	-	5.0–5.2	5.1–5.6	-	5.7–6.2	7.7–8.6	6.1–7.1
*n*	4	2	11	3	1	3	7	-	23	7	4
**Interorbital width**											
*X*‾ ± *SD*	6.4 ± 0.2**	5.7	5.4 ± 0.1	4.6 ± 0.1	4.6	4.6 ± 0.2	5.3 ± 0.1	-	6.0 ± 0.3	8.1 ± 0.3	7.1 ± 0.4
Min-max	6.2–6.7	5.6–5.8	5.3–5.6	4.6–4.7	-	4.4–4.7	5.1–5.4	-	5.6–6.9	7.6–8.4	6.5–7.3
*n*	4	2	12	3	1	3	7	-	23	7	4
**POP**											
*X*‾ ± *SD*	6.4 ± .3**	5.8	5.5 ± 0.1	4.9 ± 0.1	4.7	4.6 ± 0.3	5.3 ± 0.1	-	6.0 ± 0.2	7.9 ± 0.2	6.9 ± 0.3
Min-max	6.1–6.9	5.8–5.8	5.3–5.8	4.8–5.0	-	4.4–4.9	5.1–5.5	-	5.7–6.4	7.6–8.2	6.5–7.3
*n*	4	2	12	3	1	3	7	-	23	7	4
**POC**											
*X*‾ ± *SD*	4.8 ± 0.1**	4.7	4.8 ± 0.4	4.3 ± 0.0	4.4	4.1 ± 0.1	4.2 ± 0.2	-	4.6 ± 0.1	5.0 ± 0.2	4.3 ± 0.2
Min-max	4.7–5.0	4.5–4.8	4.5–5.9	4.3–4.3	-	4.0–4.1	3.9–4.4	-	4.2–4.8	4.6–5.2	4.1–4.5
*n*	4	2	12	3	1	3	7	-	24	7	4
**M^3^-M^3^**											
*X*‾ ± *SD*	8.0 ± 0.3**	6.5	6.0 ± 0.2	5.4 ± 0.3	5.6	5.2 ± 0.1	5.8 ± 0.2	-	6.8 ± 0.2	8.5 ± 0.2	7.7 ± 0.1
Min-max	7.5–8.2	6.4–6.5	5.8–6.2	5.2–5.7	-	5.2–5.3	5.5–6.1	-	6.6–7.2	8.3–8.7	7.6–7.9
*n*	4	2	12	3	1	3	7	-	23	7	4
**C-M^3^**											
*X*‾ ± *SD*	6.0 ± 0.2**	4.4	4.1 ± 0.1	3.9 ± 0.1	4.0	3.8 ± 0.1	4.1 ± 0.1	-	4.7 ± 0.1	6.6 ± 0.1	5.9 ± 0.2
Min-max	5.8–6.2	4.3–4.4	3.9–4.2	3.8–4.0	-	3.7–3.9	4.0–4.2	-	4.5–5.0	6.5–6.7	5.7–6.0
*n*	4	2	12	3	1	3	7	-	24	7	4
**C-C**											
*X*‾ ± *SD*	6.0 ± 0.2**	4.8	4.3 ± 0.1	3.9 ± 0.1	3.5	3.7 ± 0.1	4.4 ± 0.2	-	4.8 ± 0.2	6.4 ± 0.2	5.7 ± 0.2
Min-max	5.8–6.2	4.8–4.9	4.1–4.5	3.9–4.0	-	3.6–3.8	4.0–4.6	-	4.4–5.2	6.2–6.6	5.4–5.9
*n*	4	2	12	3	1	3	7	-	23	7	4
**Mandible**											
*X*‾ ± *SD*	12.3 ± 0.5**	9.6	9.0 ± 0.2	8.3 ± 0.2	8.2	8.6 ± 0.5	8.7 ± 0.3	-	10.1 ± 0.2	14.1 ± 0.3	12.7 ± 0.2
Min-max	11.6–12.7	9.6–9.6	8.7–9.3	8.2–8.5	-	8.2–9.1	8.4–9.1	-	9.8–10.5	13.6–14.5	12.4–12.9
*n*	4	2	11	3	1	3	7	-	24	7	4
**c-m_3_**											
*X*‾ ± *SD*	6.7 ± 0.2**	5.0	4.6 ± 0.2	4.1 ± 0.3	4.4	4.3 ± 0.4	4.5 ± 0.2	-	5.3 ± 0.2	7.5 ± 0.2	6.6 ± 0.1
Min-max	6.4–6.9	4.9–5.1	4.3–4.9	3.9–4.5	-	4.0–4.8	4.2–4.7	-	5.1–5.6	7.2–7.7	6.5–6.8
*n*	4	2	11	3	1	3	7	-	24	7	4
**Height of the upper canine**											
*X*‾ ± *SD*	2.8	2.2	1.9 ± 0.1	1.3 ± 0.1	1.4	1.2 ± 0.1	1.8 ± 0.1	-	2.2 ± 0.2	3.5 ± 0.4	3.1 ± 0.2
Min-max	-	2.1–2.2	1.7–2.1	1.2–1.4	-	1.1–1.3	1.6–1.9	-	1.8–2.4	2.9–3.9	2.9–3.4
*n*	1	2	12	3	1	3	6	-	22	7	4
**Thickness of the upper canine**											
*X*‾ ± *SD*	1.3	0.9	0.8 ± 0.2	0.7 ± 0.1	0.7	0.7 ± 0.1	0.8 ± 0	-	0.9 ± 0.1	1.6 ± 0.2	1.2 ± 0.1
Min-max	-	0.9–0.9	0.5–1.0	0.6–0.8	-	0.6–0.7	0.8–0.8	-	0.7–1.0	1.4–1.9	1.2–1.4
*n*	1	2	12	3	1	3	6	-	23	7	4
**WM^3^**											
*X*‾ ± *SD*	1.9	1.5	1.4 ± 0.1	1.3 ± 0.1	1.4	1.3 ± 0	1.4 ± 0.1	-	1.4 **±** 0.1	2.2 ± 0.2	2.0 ± 0.1
Min-max	-	1.5–1.5	1.4–1.5	1.2–1.4	-	1.3–1.3	1.3–1.4	-	1.6–2.0	2.1–2.5	1.9–2.0
*n*	1	2	12	3	1	3	7	-	24	7	4
**WM^2^**											
*X*‾ ± *SD*	2.4	1.7	1.5 ± 0.1	1.3 ± 0.1	1.5	1.3 ± 0	1.5 ± 0.1	-	1.8 ± 0.1	2.3 ± 0.1	2.3 ± 0.2
Min-max	-	1.6–1.8	1.4–1.5	1.2–1.3	-	1.3–1.3	1.4–1.6	-	1.6–2.0	2.1–2.4	2.1–2.4
*n*	1	2	12	3	1	3	7	-	24	7	4
**MRL**											
*X*‾ ± *SD*	2.9	2.1	2.1 ± 0.3	1.6 ± 0.3	2.3	1.8 ± 0.3	-	-	2.1 ± 0.2	-	-
Min-max	-	1.9–2.2	1.5–2.4	1.4–1.9	-	1.5–2.0	-	-	1.6–2.5	-	-
*n*	1	2	12	3	1	3	-	-	23	-	-
**I-M^2^ alv**											
*X*‾ ± *SD*	6.8	4.9	4.6 ± 0.2	4.3 ± 0.3	4.3	4.3 ± 0.2	4.6 ± 0.1	-	5.2 ± 0.2	7.2 ± 0.2	6.4 ± 0.1
Min-max	-	4.9–4.9	4.2–4.8	4.1–4.6	-	4.1–4.4	4.4–4.8	-	4.7–5.4	6.9–7.5	6.3–6.6
*n*	1	2	12	3	1	3	7	-	24	7	4
**Mastoid width/GLS**											
*X*‾ ± *SD*	0.57 ± 0.01**	0.63	0.65 ± 0.01	-	0.60	0.65 ± 0.01	0.63 ± 0.01	-	0.64 ± 0.01	0.56 ± 0.04	0.57 ± 0.00
Min-max	0.56–0.59	0.63–0.64	0.63–0.67	-	-	0.64–0.67	0.62–0.64	-	0.61–0.66	0.46–0.60	0.56–0.57
*n*	4	2	12	-	1	3	6	-	22	7	4
**BBC/GLS**											
*X*‾ ± *SD*	0.52 ± 0.00**	0.59	0.60 ± 0.01	-	0.56	0.61 ± 0.01	0.58 ± 0.01	-	0.58 ± 0.01	0.45 ± 0.01	0.46 ± 0.02
Min-max	0.52–0.52	0.58–0.60	0.58–0.63	-	-	0.60–0.62	0.56–0.60	-	0.55–0.61	0.43–0.46	0.46–0.47
*n*	4	2	12	-	1	3	6	-	23	7	4
**HBC/GLS**											
*X*‾ ± *SD*	0.41 ± 0.01**	0.44	0.45 ± 0.12	-	0.40	0.46 ± 0.01	0.43 ± 0.01	-	0.43 ± 0.01	0.40 ± 0.01	0.39 ± 0.02
Min-max	0.41–0.42	0.43–0.44	0.43–0.47	-	-	0.45–0.46	0.42–0.45	-	0.41–0.45	0.38–0.41	0.36–0.39
*n*	4	2	11	-	1	3	6	-	22	7	4
**ZB/GLS**											
*X*‾ ± *SD*	0.68 ± 0.01	0.71	0.71 ± 0.03	-	0.70	0.73	0.70 ± 0.02	-	0.73 ± 0.02	0.64 ± 0.02	0.67 ± 0.01
Min-max	0.67–0.69	0.71–0.71	0.68–0.77	-	-	0.72–0.75	0.67–0.73	-	0.69–0.77	0.62–0.66	0.66–0.68
*n*	4	2	10	-	1	2	6	-	22	7	4
**C-C/M^3^-M^3^**											
*X*‾ ± *SD*	0.76 ± 0.01**	0.74	0.72 ± 0.02	-	0.63	0.71 ± 0.02	0.76 ± 0.04	-	0.70 ± 0.02	0.76 ± 0.03	0.73 ± 0.03
Min-max	0.74–0.77	0.73–0.76	0.69–0.76	-	-	0.69–0.73	0.69–0.82	-	0.65- 0.75	0.72–0.80	0.70–0.77
*n*	4	2	12	-	1	3	7	-	23	7	4

* For cranial and dental measurements and ratios, significant size differences (based upon t-tests with p ≤ 0.05) between *Niumbaha* and *Glauconycteris* (all measured species combined) are indicated with **.

#### Material examined.

The collection of a new specimen of *Niumbaha superba* in South Sudan (USNM 586592) in July 2012 allowed for the examination of a live bat and for the preservation of an intact specimen in fluid. This bat was captured in a single-high ground-level mist net next to a stagnant pool of water on a rocky grasslands plateau. This plateau, located at 04°52.643'N, 027°40.557'E (elevation ~ 720 m) is surrounded by secondary thicket forest and is within the boundaries of Bangangai Game Reserve, Ezo County, Western Equatoria State. Data for previously collected specimens of *Niumbaha superba* were taken from Hayman (1939, 1947) and from Randolph L. Peterson’s notes, provided by Judith Eger at the Royal Ontario Museum. An additional specimen was recently collected in the Democratic Republic of the Congo and reported by Gembu Tungaluna (2012).

Data for *Niumbaha superba* were compared to those of various species of *Glauconycteris*, as summarized in Table 2. Additionally, for the wingtip analysis, comparisons with other, more ‘typical’ West African vespertilionids of similar size to *Niumbaha superba* (*Scotophilus leucogaster* and *Scotophilus viridis*) were made. Species/specimens examined: *Glauconycteris alboguttata* J. A. Allen, 1917 (2): Cameroon (AMNH 236329, USNM 598588); *Glauconycteris argentata* (Dobson, 1875) (14): Cameroon (AMNH 23624, AMNH 23625, AMNH 23627, AMNH 23628), Democratic Republic of the Congo (AMNH 120328, AMNH 120332, USNM 535398), Kenya (USNM 268759), Tanzania (AMNH 55545, AMNH 55546, AMNH 55548, USNM 297476, USNM 297477, USNM 297478); *Glauconycteris beatrix* Thomas, 1901 (4): Cameroon (USNM 511928, USNM 511929), Gabon (USNM 584723), Ghana (USNM 420078); *Glauconycteris curryae* Eger and Schlitter, 2001 (1): Gabon (USNM 584724); *Glauconycteris humeralis* J.A. Allen, 1917 (3): Democratic Republic of the Congo (AMNH 49014, AMNH 49312, AMNH 49315); *Glauconycteris poensis* (Gray, 1842) (12): Ivory Coast (USNM 429953, USNM 429954, USNM 429955, USNM 468192), Ghana (USNM 479528, USNM 479529, USNM 479530, USNM 479531, USNM 479533), Nigeria (AMNH 273244), Togo (USNM 437777, USNM 437778); *Glauconycteris* cf. *poensis* (6): South Sudan (new country record) (USNM 586596, USNM 586597, USNM 586598, USNM 586599, USNM 586600, USNM 586601), *Glauconycteris variegata* (Tomes, 1861) (27): Benin (USNM 421480, USNM 421481), Botswana (USNM 518696, USNM 518697), Democratic Republic of the Congo (AMNH 49060, AMNH 49061, AMNH 49062, AMNH 49063, AMNH 49066, AMNH 49067, AMNH 49068, AMNH 49070, AMNH 49195, AMNH 49313), Ghana (USNM 420077, USNM 424900), Kenya (AMNH 238490), Mozambique (USNM 304844), Nigeria (USNM 378863, USNM 378864, USNM 378865), South Africa (AMNH 257397), South Sudan (USNM 586593, USNM 586594, USNM 586595, USNM 590905), Uganda (AMNH 184228); *Niumbaha superba* (Hayman, 1939) (4): Democratic Republic of the Congo (RMCA 14.765), Ivory Coast (RMCA A9363), Ghana (BMNH 47.10), South Sudan (USNM 586592); *Scotophilus leucogaster* (Cretzschmar, 1830) (8): Benin (USNM 421421, USNM 421424, USNM 421425), Burkina Faso (USNM 450698, USNM 452887, USNM 452889, USNM 503955), Sierra Leone (USNM 547030); *Scotophilus viridis* (Peters, 1852) (9): Ivory Coast (USNM 468194, USNM 468195, USNM 468199), Mozambique (USNM 365411, USNM 365412, USNM 365413, USNM 365414, USNM 365417, USNM 365418). Museum abbreviations and information: USNM: National Museum of Natural History, Smithsonian Institution, (Washington, D.C., USA); AMNH: American Museum of Natural History (New York, USA); BMNH: British Museum of Natural History (London, UK); RMCA: Royal Museum for Central Africa (Tervuren, Belgium).

#### Notes.

Species of *Glauconycteris* are quickly recognized by a variety of distinctive traits, many of which are shared with the monotypic *Niumbaha*. Below we examine each of these traits, highlighting similarities and differences between *Niumbaha* and *Glauconycteris*.

*Coloration, pattern, and body size*: Hayman (1939) described and illustrated the coloration and patterning of this bat in detail, based upon the first specimen collected in Belgian Congo (now Democratic Republic of the Congo) (RMCA 14.765). He noted the presence of: (1) two sets of stripes on the dorsum - one set of “lanceolate stripes” found on each side of the median dorsal line of the back starting near the base of the neck and tapering to an end near the middle of the back, and one set of longer, narrower stripes on either side of the body, each commencing a little in advance of and lateral to the ends of medial stripes and each terminating just short of the root of the tail; (2) a set of stripes that begin on the dorsal side of each shoulder and run over the shoulder to the venter where they widen and run the lateral length of the venter joining and widening in the perineal region; (3) a wide throat band that connects to the shoulder/venter stripe, and (4) three spots – one roughly circular patch on the top of the muzzle between the eyes and one at each side of the face at the base of each ear.

In 1947, Hayman described the second specimen collected, this time from the Gold Coast (Ghana) (BMNH 47.10). Hayman found the markings of this specimen sufficiently different from the holotype of *superba* that he erected a new subspecies based upon it, *Glauconycteris superba sheila*. The patterning of this specimen differs in that (1) two white spots are found on each shoulder next to the base of the humerus, (2) the unpigmented areas on the upper surface of the elbow, knee and ankle joints are present, and (3) the ventral interfemoral membrane is a pale gray color. Our newly collected specimen more closely resembles the Ghana specimen, but has only one white spot on each shoulder next to the base of the humerus and lacks an unpigmented area at the base of the ankle (Fig. 2). The recent DRC specimen (Gembu Tungaluna 2012) resembles our South Sudan specimen, but has the unpigmented ankle spots. The only other specimen of *Niumbaha superba* is from the Ivory Coast (RMCA A9363) and, while cited by Peterson and Smith (1973), it has not been described in the literature and we have not examined it. However, Peterson, in his museum notes, noted that it corresponds to *Glauconycteris superba sheila* (Peterson, in litt., Royal Ontario Museum notes). Thus, of the five specimens, four appear to have characteristics attributed to the subspecies *sheila* and only one to the nominate subspecies. However, given the variation seen within the specimens of the subspecies *sheila* and giventhat the single specimen attributed to the nominate subspecies was captured in relatively close proximity to two specimens that match more closely the pelage patterning described for *sheila*,we do not recognize *sheila* as a valid subspecies (see also Simmons 2005). Within species of *Glauconycteris*, the tendency to produce patterns of spots, stripes and reticulations is pronounced and variable (Rosevear 1965). In *Glauconycteris poensis*, for example, Hayman and Jones (1950) described “remarkable” variation in the pattern of white shoulder spots and flank stripes, suggesting that variation is normal for this and related species. Further study, ideally based upon the collection and (morphological and genetic) study of additional material from additional localities, will be needed to ascertain whether clear patterns of geographic variation exist within *Niumbaha superba* and whether multiple subspecies can be recognized.

Notably, our specimen of *Niumbaha superba* (and that reported by Gembu Tungaluna 2012) was *not* originally black and white when collected, but rather black and cream/buffy yellow. Hayman (1939, 1947) described *superba* from museum specimens, in which we suspect the color had faded (Rosevear [1965] also noted the “pure white hairs” and included a drawing of *Glauconycteris superba sheila*, taken from a black and white photograph [from which the original color is thus not clear] of the bat on a tree trunk). Indeed, our specimen, fixed in formalin and stored in ethanol, is now black and white, such that the yellow coloration of the paler fur ornamentation has leached from the fur, and only the images of the freshly collected bat indicate its true color.

Finally, *Niumbaha superba* is larger than all species of *Glauconycteris*, as noted by Hayman (1939, 1947). Rosevear (1965) subsequently noted the larger body size as well, but also noted that body size measurements are not “very much larger” than *Glauconycteris variegata* and *Glauconycteris argentata*, but that the skull is far bigger, with a total skull length greater than 16mm (Table 2; see also discussion below).

*Wing morphology*: Rosevear (1965) distinguished *Glauconycteris* from other African Vespertilioninae by its distinctive wing morphometry – noting that phalanx 2 (Ph2) on digit 3 (DIII) is longer than Ph1. Within *Glauconycteris*, *Glauconycteris variegata* is perhaps the best studied species and Findley et al. (1972) described it being among the bat species with the highest aspect ratio (wing length/wing width) and the longest wing tips. Wing size and shape represent a compromise between different (and often conflicting) selective forces and the kinematics of bat flight are complex (Norberg and Rayner 1987). Nevertheless, we can say that the long pointed wingtips and high aspect ratio of *Glauconycteris variegata* suggest relatively maneuverable, low flight speed that might favor feeding in open areas around, but not within clutter (Norberg and Rayner 1987; and see Obrist et al. 1989, whose examination of echolocation calls also supported this flight/feeding mode). *Niumbaha superba*, while retaining Ph2>Ph1 for DIII, diverges from *Glauconycteris* in that the ratio of Ph2/Ph1 is significantly less extreme (1.15 ± 0.05 SD vs. 1.51 ± 0.12 SD; t = -6.12, df = 31, p < 0.0001; Fig. 4), which has not previously been noted for this taxon. This suggests that *Niumbaha* is perhaps closer to ‘typical’ vespertilionids in ecomorphological space (for comparison, measurements for *Scotophilus* are also included in Fig. 4). This difference in wing shape may reflect differences in habitat type and feeding mode (see also the discussion of differences in dentition between *Niumbaha* and *Glauconycteris*, below).

*Facial features (including the ear)*: *Glauconycteris* is distinctive among African vespertilionids in possessing an extremely shortened but broad muzzle in which the nostrils open more or less to the side from a transverse, thick subcylindrical naked pad. On the underlip is found a thickened pair of pads and the lower lip near the corner of the mouth has a fleshy lappet or fold that can be made to extend horizontally (Rosevear 1965). The rostrum is proportionally longer in *Niumbaha superba* as compared to *Glauconycteris*, but we have found no mention in the literature of differences in other facial features.We note here that the fleshy lappet is present on the lower lip but that the muzzle appears to be more robust and contains nostrils that open more to the front than to the side (Fig. 3), a more ‘typical’ vespertilionid configuration.

The ears of *Glauconycteris sensu stricto* are of small to moderate size and rounded with a strong semicircular inner margin that ends basally in a “curiously backwardly projecting lobe” and a pronounced antitragus (Rosevear 1965:273). The tragus is “sickle” or half-moon shaped with a large and broadly triangular basal lobe. In his original description of *Niumbaha superba*, Hayman (1939) noted that the ears are less rounded and more subquadrangular than in other *Glauconycteris* (Fig. 3). Rosevear (1965:284-285), noting that his observations were from a dried skin, described the inner margin of the ear of *Niumbaha superba sheila* as “terminating in a long almost parallel-sided free lobe”, the antitragus as large and semicircular, and the tragus as broader than in other *Glauconycteris* with a “boldly curved” outer margin and a small acute lobule. Based upon examination of the fresh and subsequent fluid specimen from South Sudan, we generally concur. The “free lobe” at the inner margin of the ear is larger in *Niumbaha* than in *Glauconycteris*, but we note that the antitragus is more squared off than semicircular. Additionally, the horizontal cartilaginous ridges in the outer ear margin are pronounced in *Niumbaha* (especially in the fresh specimen; Fig. 3) relative to *Glauconycteris*.

*Cranial features*: Despite placing this bat in *Glauconycteris*, Hayman (1939:222) noted that the skull was longer and less broad with marked flattening of the rostrum “so that the profile shows an angle at the junction of the brain-case and the rostrum” and (1947:549) and so that there is “considerable lengthening of the infraorbital foramen”; he also noted the presence of proportionally deeper basisphenoid pits (Fig. 5). Rosevear (1965) noted that the skull is significantly larger and more powerful than *Glauconycteris sensu stricto* and that the upper surface of the rostrum does not rise in an even plane from the incisors to the occiput (as occurs in most *Glauconycteris*, see skull images of *Glauconycteris variegata* and *Glauconycteris poensis* in Fig. 5) but rather is flat or roughly parallel to the upper toothrow. This results in an excavation or “hollowing-out” of the frontal region of the skull (Fig. 5). Lastly, while *Glauconycteris* have a domed braincase with virtually no sagittal crest, a low crest is present in *Niumbaha*, where it joins posteriorly with a lambdoidal crest to form a low supraoccipital pyramid (Rosevear 1965).

*Niumbaha* shares its dental formula and many dental characteristics with *Glauconycteris*. The dental formula is 2.1.1.3/3.1.2.3 = 32, but Hayman (1939) noted a greater proportional difference in size between the lower i_1_ and i_3_ than in *Glauconycteris sensu stricto* (Fig. 5). As with *Glauconycteris*, the upper incisor is long and pointed and the upper premolar is long, similar in height to the molars. While Hayman (1947) noted a considerably reduced m_3_ compared to other (we presume *Glauconycteris*) species, we do not find this to be the case in our South Sudan specimen. The canines, and especially the upper canine, are considerably more robust (unreduced) in *Niumbaha* than in *Glauconycteris*. The size difference between *Niumbaha* and *Glauconycteris* presumably allows *Niumbaha* to take larger, more hard-bodied prey than *Glauconycteris*, an apparent lepidopteran (moth) specialist (Fenton et al. 1977).

**Figure 6.**
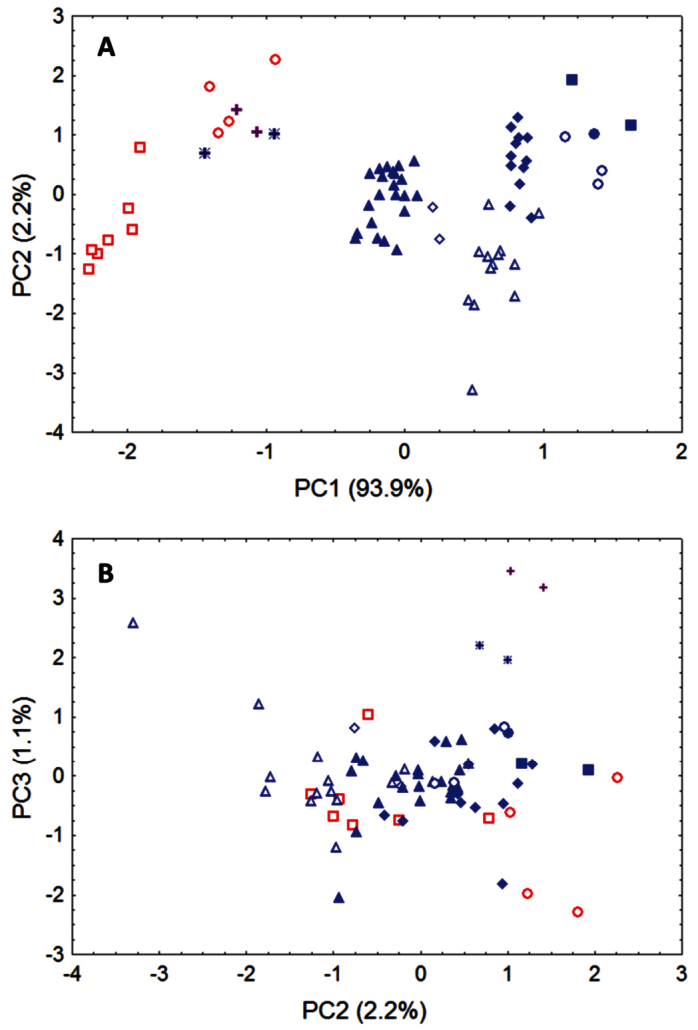
Morphometric separation (first three principal components of a Principal Components Analysis) of 12 cranial and dental measurements. Data are from 70 adult skulls of *Glauconycteris*, *Niumbaha*, and *Scotophilus* (with measurements following Table 1 and 2). Specimens of *Scotophilus*, included for ecomorphological comparison, are indicated in red (**open red squares**, *Scotophilus leucogaster*; **open red circles**, *Scotophilus viridis*). Specimens of *Glauconycteris* are indicated in blue (**open blue diamonds**, *Glauconycteris alboguttata*; **open blue triangles**, *Glauconycteris argentata*; **open blue circles**, *Glauconycteris beatrix*, **closed blue circles**, *Glauconycteris curryae*; **closed blue squares**, *Glauconycteris humeralis*; **closed blue diamonds**, *Glauconycteris poensis*; **closed blue triangles**, *Glauconycteris variegata*). Specimens of *Niumbaha superba* from central Africa (DRC, S Sudan) are marked with **crosses**; specimens of *Niumbaha superba* from west Africa (Cote D’Ivoire, Ghana) are marked with **asterisks**. **A** Skulls of *Niumbaha* separate from skulls of species of *Glauconycteris* in combination along the first and second components, suggesting greater overall ecomorphological resemblance of *Niumbaha* with medium-sized, less specialized African vespertilionids such as *Scotophilus*. The first principal component reflects distinctions in overall skull size, which increases from right to left. **B** Separation of skulls of *Niumbaha* from those of *Glauconycteris* and *Scotophilus* in combination along the second and third components indicates the morphological isolation of *Niumbaha* and illustrates consistent differences in skull shape, reflecting (in separation along the third component) the proportionally narrower interorbital dimensions, less dramatic postorbital constriction, longer toothrows, narrowed skull, but widened anterior rostrum in *Niumbaha* relative to *Glauconycteris*.

Our principal components analysis of cranial and dental data (based upon measurements listed in Table 2 from *Niumbaha*, *Glauconycteris*, and *Scotophilus*) clearly indicates that the skulls of *Niumbaha* separate from skulls of species of *Glauconycteris*, suggesting greater overall ecomorphological resemblance of *Niumbaha* with medium-sized, less specialized African vespertilionids such as *Scotophilus* (Fig. 6). The first principal component reflects distinctions in overall skull size and indeed each of the cranial measurements in this analysis is significantly larger for *Niumbaha* than for *Glauconycteris* (see Table 2). Beyond size, separation of skulls of *Niumbaha* from those of *Glauconycteris* and *Scotophilus* in combination along the second and third components indicates the morphological isolation of *Niumbaha* and illustrates consistent differences in skull shape, reflecting (in separation along the third component) the proportionally narrower interorbital dimensions, less dramatic postorbital constriction, longer toothrows, narrowed skull, but widened anterior rostrum in *Niumbaha* relative to *Glauconycteris*.

**Table 3.** Factor loadings, eigenvalues, and percentage of variance explained by illustrated components (Fig. 6) from Principal Components Analysis of 70 adult skulls of *Glauconycteris*, *Niumbaha*, and *Scotophilus*. Principal components were extracted from a covariance matrix of 12 log-transformed cranial measurements (see Table 1, 2).<br/>

**Variable**	**PC1**	**PC2**	**PC3**
Zygomatic breadth	-0.988	0.003	-0.044
Mastoid width	-0.962	-0.083	-0.098
Breadth of braincase	-0.940	-0.218	0.082
Height of braincase	-0.969	-0.137	-0.020
Interorbital width	-0.970	-0.109	-0.160
Postorbital process width	-0.971	-0.133	-0.146
Postorbital constriction	-0.489	-0.726	0.449
Width at M^3^	-0.977	0.035	0.064
Maxillary toothrow length (C-M^3^)	-0.985	0.129	0.073
Width at upper canines	-0.966	0.054	0.091
Greatest length of mandible	-0.989	0.077	0.012
Mandibular toothrow length	-0.983	0.130	0.054
Eigenvalues	0.222	0.005	0.003
Percent variance (%)	93.9	2.1	1.1

#### Distribution and habitat.

**Figure 7.**
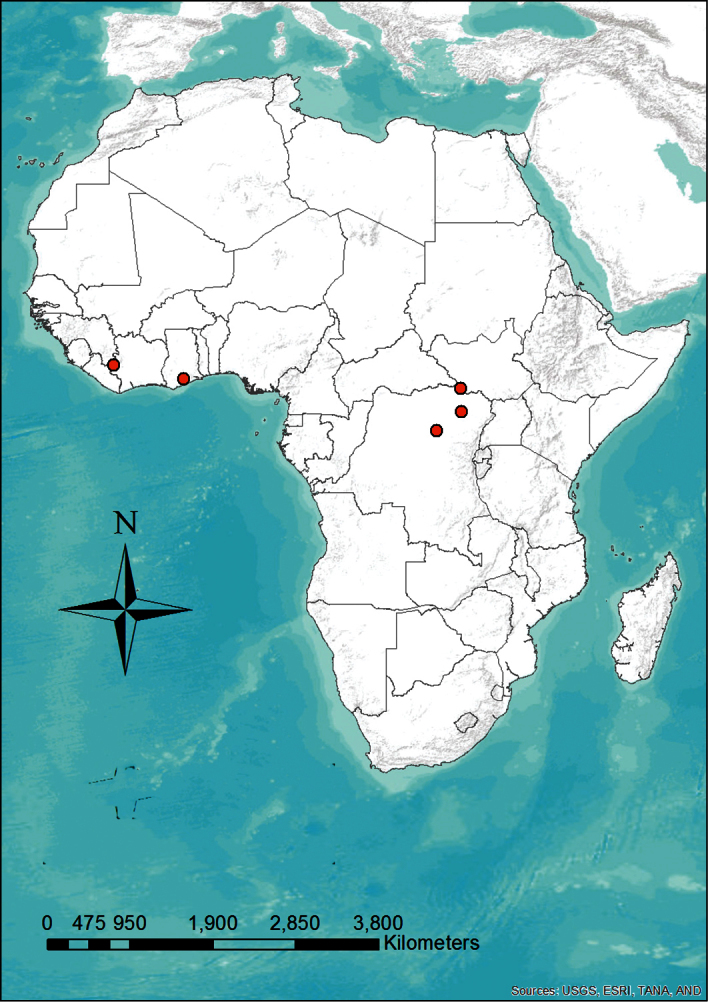
Distribution map showing the locations of the five recorded specimens of *Niumbaha superba*. Given how widely distributed this species is, its rarity in collections is enigmatic.

*Niumbaha superba* has been rarely captured (only five times) but is apparently widely distributed (Fig. 7), being recorded from Ghana, Ivory Coast, Democratic Republic of the Congo and South Sudan. This broad distribution suggests that it is more common than its collection records indicate. Although most species in its apparent sister genus, *Glauconycteris*,are not well known, at least one species (*Glauconycteris variegata*) is believed to be a high flier (Obrist et al. 1989), which could translate to poor capture success for *Niumbaha*, especially if it typically flies at even greater heights. *Glauconycteris* are found in a variety of habitats, mostly from moist forest zones (Rosevear 1965). We can only speculate that *Niumbaha* is found in similar habitat types. Neither the description of the first specimen collected in the Democratic Republic of Congo (Hayman 1939) nor that of the second specimen from Ghana, which was “found alive on the ground” (Hayman 1947:550) contain habitat descriptions. However, Rosevear (1965) noted that both locations were in closed forest (though the Ghana location was on the edge of closed forest and a Guinea woodland zone) and Hayman and Hill (1971) noted that both locations are from heavy rain forest. A recent specimen from Democratic Republic of the Congo was mist-net captured in secondary forest (Gembu Tungaluna 2012) and our specimen from South Sudan was mist-net captured on a grassland plateau just above a secondary thicket forest.

## Discussion

The generic placement of “*Glauconycteris” superba* has never been critically reviewed. Only four specimens have previously been mentioned in the literature (Hayman 1939, 1947; Peterson and Smith 1973; Gembu Tungaluna 2012), with minimal comment on the distinctness of this species from other *Glauconycteris* in cranial features, nostril and ear anatomy, and wing proportions (in addition to differences in skull size, robusticity, and pelage patterning, which have been noted previously). Very few reviewers of *Glauconycteris* have mentioned first-hand examination of specimens of *superba* or their attributes. Obviously, it is not only on the basis of its ecomorphological distinction from other species of *Glauconycteris*, but especially in its lack of several of the most characteristic morphological properties of *Glauconycteris* (which we take to be synapomorphic for the species of *Glauconycteris sensu strict*o), that we erect a new genus, *Niumbaha*, to house *superba*, one of the most beautiful and rarely collected of Africa’s vespertilionids. In lacking the reduced body size, extremely blunt face, characteristic nostril configuration, and extreme wingtip lengthening of *Glauconycteris*, *Niumbaha* superficially reminds us of other medium-sized and less specialized vespertilionid genera, such as *Scotophilus* (Fig. 6). We advocate integrating DNA sequence data for *Niumbaha superba*, and for as wide a sampling of species of *Glauconycteris* as possible, into current phylogenetic datasets and frameworks for African vespertilionid bats (Hoofer and Van Den Bussche 2003; Roehrs et al. 2011), to test our hypothesis that *Niumbaha* lies outside the phylogenetic scope of *Glauconycteris sensu strict*o.

Our naming of a new genus for one of the most extraordinary and rarest-collected bats in Africa highlights a number of issues. *Niumbaha superba* displays one of the most striking pelage patterns known in bats. While species of *Glauconycteris* are known for their spots, stripes, and wing reticulation, none are so boldly patterned as *Niumbaha superba*. Similar markings are found in only a small number of vespertilionids, especially the East Asian Harlequin bat, *Scotomanes ornatus*, and the western North American Spotted bat, *Euderma maculata*, as well as in (albeit to a considerably lesser extent) some emballonurids, such as *Saccopteryx bilineata*. Rosevear (1965:285) noted that “though the bold black and white coloring of [*Niumbaha superba*] … may appear very conspicuous in the hand it doubtless acts as a concealing pattern in nature in a similar way to that well-established for many other animals with disruptive markings…” Such disruptive coloration may, in part, explain the lack of local and scientific knowledge regarding this bat. In each collection location it was unknown to indigenous peoples, and early scientific collecting of bats was often focused on areas where they could be most obviously located, such as buildings or other conspicuous roost locations. Santana et al. (2011) studied relationships between bat roosting habitats and the presence of stripes, throat bands and spots, and demonstrated the independent evolution of pelage markings in 12 of 19 families of bats studied. In particular, they noted an association between roosting in vegetation (especially tent making) and the evolution of stripes and neckbands. They added that crypsis through disruptive stripes and neck bands could be augmented by facial markings (as occur in several tent-making species) and that this crypsis could be enhanced by blending with the patterns of light and shadows created by sunlight peeking through small gaps in the leaf tents. There are no documented examples of tent-making in African bats, although it has arisen independently on other continents (Santana et al. 2011). The possibility that *Niumbaha superba* might be a tent-making bat is intriguing. Another possibility is that the striking pelage pattern of *Niumbaha superba* is not disruptive or camouflaging, but rather serves in social signaling. However, the use of pelage markings (outside of epaulettes) as social signals in bats is not well studied (Santana et al. 2011) and the apparent lack of sexual dimorphism in the pattern of *Niumbaha superba* suggests that their coloration may not play a social role. Similarly, it is possible that *Niumbaha superba*’s pattern and coloration is aposematic, but this is otherwise unknown in bats (Caro 2005). Strong chemical defenses are associated with some other boldly patterned black and white mammals (e.g., mephitids, mustelids such as *Ictonyx* and striped possums such as *Dactylopsila*), but we did not detect a strong scent in our specimen. Regarding its common name, *Niumbaha superba* was originally described by Hayman (1939) as resembling the spotted skunk *Spilogale* and has had several common names over the years, including the Magpie bat (Hayman 1947), Mrs. Cansdale’s bat (Hayman 1947), and Pied bat (Wilson and Cole 2000, as *Chalinolobus superba*). Given that several species of the Australo-Papuan genus *Chalinolobus* are referred to as ‘pied bats’, we think it best to avoid that name, and propose the use of ‘badger bat’ in reference to its tenacious appearance and its bold black and white/cream coloration, both reminiscent of badgers.

The conservation status of poorly known species such as *Niumbaha superba* is difficult to assess. Until 2004, the International Union for the Conservation of Nature (IUCN) listed *Niumbaha superba* as “Vulnerable”. In 2008, it changed the listing to “Least Concern” “in view of its wide distribution, presumed large population, and because it is unlikely to be declining fast enough to qualify for listing in a more threatened category” (Fahr et al. 2008). We concur, especially in light of the two 2012 captures. Nevertheless, any detailed understanding of the current status of this bat will require considerable further study.

The capture of this bat in South Sudan (as well as the collection of *Glauconycteris* cf. *poensis*, a new country record) highlights the need to expand biodiversity surveys and studies in this new nation. These bats were captured in the Bangangai Game Reserve in Western Equatoria State, which resides within a ‘tropical belt’ along the border with the Democratic Republic of the Congo. It is largely composed of dense tropical/subtropical forest, the type of which is highly restricted in South Sudan. Its placement near the Congo Basin ecoregion sets it apart from the rest of South Sudan and elements of the faunas and floras of West Africa and East Africa overlap here (Linder et al. 2012), creating significant biodiversity. Koopman (1975) in his seminal work on the bats of Sudan, highlighted the need to survey for bats in this unstudied region.

In his original description of *Niumbaha superba*, Hayman (1939:223) concluded “that such a conspicuous new species should be found in a region which has received considerable attention from museum collectors of proved ability … is somewhat surprising. It seems that much more collecting needs to be done before we can claim a complete knowledge of the mammalian fauna of tropical Africa.” More than 70 years later, this statement still holds, and the biota of many areas of sub-Saharan Africa remains poorly understood, even in vertebrate groups usually considered well studied, such as mammals (Reeder et al. 2007). As an understanding of basic biodiversity is the backbone upon which other studies and conservation programs can be built, we encourage further basic field and museum work in the region; many more surprises no doubt await.

## Supplementary Material

XML Treatment for Niumbaha
